# Protective effect of Soluble Epoxide Hydrolase Inhibition in Retinal Vasculopathy associated with Polycystic Kidney Disease

**DOI:** 10.7150/thno.43154

**Published:** 2020-06-22

**Authors:** Jihong Lin, Jiong Hu, Andrea Schlotterer, Jing Wang, Matthias Kolibabka, Khader Awwad, Nadine Dietrich, Kristin Breitschopf, Paulus Wohlfart, Aimo Kannt, Katrin Lorenz, Yuxi Feng, Rüdiger Popp, Sigrid Hoffmann, Ingrid Fleming, Hans Peter Hammes

**Affiliations:** 1 5th Medical Department, Medical Faculty Mannheim, University of Heidelberg, D-68167 Mannheim, Germany.; 2 Institute for Vascular Signalling, Center for Molecular Medicine, Goethe University, D-60590 Frankfurt, Germany.; 3 Sanofi Research and Development, Industriepark Hoechst, D-65926 Frankfurt, Germany.; 4 Institute of Experimental and Clinical Pharmacology and Toxicology, Medical Faculty Mannheim, University of Heidelberg, D-68169 Mannheim, Germany.; 5 Center of Medical Research, Medical Faculty Mannheim, University of Heidelberg, D-68167 Mannheim, Germany.; 6 Center of Medical Research, Medical Faculty Mannheim, University of Heidelberg, D-68167 Mannheim, Germany.

**Keywords:** retinal vasoregression, sEH inhibition, CD74, 19, 20-DHDP, neurovascular unit

## Abstract

**Rationale:** Vasoregression secondary to glial activation develops in various retinal diseases, including retinal degeneration and diabetic retinopathy. Photoreceptor degeneration and subsequent retinal vasoregression, characterized by pericyte loss and acellular capillary formation in the absence diabetes, are also seen in transgenic rats expressing the polycystic kidney disease (PKD) gene. Activated Müller glia contributes to retinal vasodegeneration, at least in part via the expression of the soluble epoxide hydrolase (sEH). Given that an increase in sEH expression triggered vascular destabilization in diabetes, and that vasoregression is similar in diabetic mice and PKD rats, the aim of the present study was to determine whether sEH inhibition could prevent retinal vasoregression in the PKD rat.

**Methods:** One-month old male homozygous transgenic PKD rats were randomly allocated to receive vehicle or a sEH inhibitor (sEH-I; Sar5399, 30 mg/kg) for four weeks. Wild-type Sprague-Dawley (SD) littermates received vehicle as controls. Retinal sEH expression and activity were measured by Western blotting and LC-MS, and vasoregression was quantified in retinal digestion preparations. Microglial activation and immune response cytokines were assessed by immunofluorescence and quantitative PCR, respectively. 19,20-dihydroxydocosapentaenoic acid (19,20-DHDP) mediated Notch signaling, microglial activation and migration were assessed *in vivo* and *in vitro*.

**Results:** This study demonstrates that sEH expression and activity were increased in PKD retinae, which led to elevated production of 19,20-DHDP and the depression of Notch signaling. The latter changes elicited pericyte loss and the recruitment of CD11b^+^/CD74^+^ microglia to the perivascular region. Microglial activation increased the expression of immune-response cytokines, and reduced levels of Notch3 and delta-like ligand 4 (Dll4). Treatment with Sar5399 decreased 19,20-DHDP generation and increased Notch3 expression. Sar5399 also prevented vasoregression by reducing pericyte loss and suppressed microglial activation as well as the expression of immune-response cytokines. Mechanistically, the activation of Notch signaling by Dll4 maintained a quiescent microglial cell phenotype, i.e. reduced both the surface presentation of CD74 and microglial migration. In contrast, in retinal explants, 19,20-DHDP and Notch inhibition both promoted CD74 expression and reversed the Dll4-induced decrease in migration.

**Conclusions:** Our data indicate that 19,20-DHDP-induced alterations in Notch-signaling result in microglia activation and pericyte loss and contribute to retinal vasoregression in polycystic kidney disease. Moreover, sEH inhibition can ameliorate vasoregression through reduced activity of inflammatory microglia. sEH inhibition is thus an attractive new therapeutic approach to prevent retinal vasoregression.

## Introduction

Glial cells mediate the crosstalk between neurons and vascular cells, and in particular communication within the retinal neurovascular unit 1. Retinal neurodegeneration is accompanied by glial activation and vasoregression, which is characterized by pericyte loss and subsequent acellular capillary formation 2-4, as demonstrated in a model of ciliopathy-associated retinal degeneration 5, 6. Ciliopathy is a group of human genetic disorders such as Bardet-Biedl syndrome, polycystic kidney diasease (PKD) and retinitis pigmentosa, caused by the abnormal formation or function of cellular cilia. Ciliopathies often share common features such as retinal degeneration, brain anomalies, cognitive impairment, renal and liver dysfunction. Approximately 10-30 % of patients that suffer from ciliopathies, e.g. Senior Loken syndrome, show signs of retinal degeneration (retinitis pigmentosa). Also, mutations in nephrocystin 1-6 7 and rhodopsin cause autosomal dominant retinitis pigmentosa 8. The PKD rat is a model of induced ciliopathy arising from a transgene that overexpresses a truncated human polycystin-2 gene in the kidney, large and small intestine, pancreas and photoreceptors in the retina 6, 9. In this model, vasoregression starts between 4 and 8 weeks after birth, and is accompanied by microglia activation. Müller glia undergo reactive gliosis and multiple cellular changes, including the redistribution of aquaporins and altered expression of the glial cell-specific potassium channel Kir4.1 10-13. Microglial activation in PKD rats is reflected by the upregulation of CD74 14, and CD74 positive cells can be detected in close proximity to retinal capillaries undergoing vasoregression, suggesting a causal link between the two processes 15.

Microglial activation and migration as well as polarization are regulated by Notch signaling and ω-3 polyunsaturated fatty acids (PUFA), such as docosahexaenoic acid (DHA), which are highly enriched in the retina 16-18. DHA is a major structural lipid of the outer segment membranes of the photoreceptor 19, and low DHA levels have been linked with retinal neurodegeneration 20, 21. Although the dietary supplementation of DHA can prevent retinal degeneration in rats 22, 23, an effect that has been linked to Notch signaling 24, detrimental effects have also been observed in diabetic rats treated with PUFA 25. At present, it is also not clear whether the effects of DHA are due to the parent lipid itself or DHA-derived metabolites generated by the sequential action of cytochrome P450 (CYP) enzymes and the soluble epoxide hydrolase (sEH). For example, the diol 19,20-dihydroxydocosapentaenoic acid (DHDP) is generated by the sEH from the epoxide 19,20-epoxydocosapentaenoic acid (19,20-EDP), which is in turn generated by CYP enzymes from DHA. This is relevant as 19,20-DHDP, generated by Müller glia cells, regulates physiological angiogenesis via the direct inhibition of the γ-secretase 26, which is involved in the cleavage and activation of Notch signaling 27. More recently, however, much higher concentrations of the same metabolite were detected in diabetic human and murine retinas and linked with pericyte loss, acellular capillary formation and breakdown of the blood retinal barrier (BRB). Inhibition of the sEH prevented both, vasoregression and BRB breakdown in a mouse model of diabetic retinopathy 28. Given that an increase in sEH expression triggered vascular destabilization 29, and that vasoregression is also a characteristic of the retinal phenotype in PKD rats, the aim of the present study was to determine whether or not sEH inhibition could prevent or interfere with retinal vasoregression in the PKD rat.

## Materials and Methods

### Animals

Transgenic PKD rats were generated at the center of medical research, medical faculty Mannheim, University of Heidelberg. A 2.2-kbp cDNA fragment that encoded the truncated human polycystin-2 protein was subcloned into a pUCTrans vector under the cytomegalovirus (CMV) promoter 6. Animals were held in a 12-hours light and dark cycle with free access to food and drinking water. For this study, a sEH inhibitor (sEH-I) (Sar5399, Sanofi, Germany; International Publication Number: WO 2014/111465 A1, International Application Number: PCT/EP2014/050796) or vehicle was administered to one month old male homozygous PKD rats through daily intraperitoneal injections (i.p.), at a dose of 30 mg/kg body weight, over a period of four weeks. The wild-type littermate Sprague-Dawley (SD) rats, received solvent as control group. After treatment, rats were anesthetized and sacrificed. The eyes were enucleated and either fixed in 4% formalin for retinal digestion or frozen at minus 80°C for protein and RNA isolation. All experiments were approved by the governmental authorities (animal license numbers 35-9185.81/G-219/10).

### Cryosection immunohistochemistry

Eyes were embedded in OCT compound (Tissue Tek/Sakura, Staufen, Germany) and cut into 8 µm thick sections. After fixation, retinal sections were blocked and permeabilized in 1% BSA and 0.5% Triton X-100 overnight at 4°C. Then, retinal sections were incubated with primary antibody against sEH (1:250, provided by Prof. Michael Arand, Zurich, Switzerland) 30 and GFAP (1:1000, Millipore, Hamburg, Germany). Alexa-Fluor-488 and Alexa-Fluor-594 (both Invitrogen, Darmstadt, Germany) conjugated secondary antibodies were used for fluorescent signaling detection. Rabbit IgG (1:250, sc-2027, Santa Cruz, Germany) served as negative control for sEH.

### sEH activity measurement and LC-MS/MS PUFA epoxide and diol profiles

sEH activity assay: Retinas from SD and PKD rats were homogenized at 4°C in lysis buffer containing 50 mmol/L Tris-HCl pH 7.5, 150 mmol/L NaCl, 10 mM sodium pyrophosphate (NaPPi), 20 mmol/L NaF, 1% sodium deoxycholate, 1% Triton X-100 and 0.1% SDS. For determination of the sEH activity 5 μg protein of the homogenate were incubated at 37 °C for 10 minutes in 100 μL of potassium phosphate buffer (100 mmol/L, pH 7.2). Reactions were started by the addition of the substrate 14,15- epoxyeicosatrienoic acid (14,15-EET) (10 μmol/L) in presence of sEH-I (10 µmol/L) or DMSO, stopped on ice and immediately extracted twice with ethyl acetate (0.7 mL). The production of 14,15-dihydroxyeicosatrienoic acids (14,15-DHET) were quantified by LC-MS/MS. For liquid chromatography-tandem mass spectrometry (LC-MS/MS) analysis, one-tenth of the sample was spiked with a deuterated internal standard (14,15-EET-d8). After evaporation of the solvent in a vacuum block under a gentle stream of nitrogen, the residues were reconstituted with 50 μL of methanol:water (1:1, v/v) and determined with a Sciex mass spectrometer (AB Sciex, Darmstadt, Germany) operating in multiple reaction monitoring (MRM) mode as previously described 26, 31. Chromatographic separation was performed on a Gemini C18 column (150 × 2 mm inner diameter; 5 μm particle size; Phenomenex, Aschaffenburg, Germany). sEH activity was assessed by determining the generation 14,-15 DHET per µg protein/minute.

Fatty acid epoxide/diol profiling: Retina homogenate (5 µg) or 100 µL serum samples were used for LC-MS measurement. All fatty acid epoxides and diols including 5,6-EET, 5,-6-DHET, 8,9-EET, 8,9-DHET, 11,12-EET, 11,12-DHET, 14,15-EET, 14,15-DHET, 19,20-EDP and 19,20-DHDP were obtained from Cayman Europe (Hamburg, Germany).

### Retinal digest preparation

Eyes were obtained from sEH-I or vehicle treated homozygote PKD rats and their wild-type littermates (n=5 for each group). Retinal vascular preparations were performed using a trypsin digestion technique as previously described 4, 32. The eyes were fixed in 4% PFA for at least 2 days. Retinae were isolated from the eye-cup and washed in distilled water at 37°C for 1 hour, then transferred to a solution of 3% trypsin dissolved in 0.2 mol/L Tris-HCl (pH 7.4) and digested for 3 hours at 37°C. After intensive washing under a dissection microscope, the retinal vessel nets were dried on a clean glass slide overnight and stained with periodic acid Schiff's regent (PAS) as well as haematoxylin to highlight basement membranes of retinal capillaries and nuclei.

### Retinal quantitative morphometry

Acellular capillaries were assessed with PAS-haematoxylin stained retinal vasculature and by using an integrated ocular with 400 times magnification, normalized to relative retinal area (AC number/mm^2^ retinal area). Pericyte and migrating pericyte number were quantified relative to the retinal capillary area (cell number/mm^2^ capillary area) in ten randomly selected fields (400× magnification) using an image analysis system of Cell^F^ (Olympus Opticals, Hamburg, Germany). Pericytes, migrating pericytes and endothelial cells were defined according to morphology as described in detail elsewhere 33, 34.

### Whole mount immunohistochemistry and microglia quantification

Whole mount retinae were fixed in 4% PBS-buffered formalin for 3 hours at room temperature. After washing, retinae were blocked and permeabilized in 1% BSA and 0.5% Triton X-100 overnight at 4°C. Following three washes with PBS buffer (pH 6.8) containing 0.5% Triton X-100, 1 mmol/L CaCl_2_ and 1 mmol/L MnCl_2_, the retinae were incubated with biotin conjugated isolectin B4 (1:100, Sigma-Aldrich, L1240, Taufkirchen/Munich, Germany) overnight. Primary antibodies against CD11b (mouse anti-rat, AbD SeroTec, MCA275G, 1:100 dilution) and CD74 (rabbit anti-rat, Santa Cruz, SC-20082, 1:100 dilution) were diluted in blocking buffer as above and incubated at 4°C overnight. The secondary antibodies porcine anti-rabbit TRITC (DAKO, R0156, 1:20 dilution), goat anti-mouse Alexa Fluor^®^ 488 (Invitrogen, A21131, 1:200 dilution), and streptavidin Alexa Fluor^®^ 633 (Invitrogen, S21375, 1:500 dilution) were incubated at room temperature for one hour. All images were scanned using Leica TCS SP2 confocal microscope. Microglia were defined as expressing CD11b^+^ and CD74^+^ and quantified in ten randomly selected fields (400x magnification) from superficial and deep vascular retinal layers.

### Western blotting

Retinae were dissected and homogenized on ice in lysis buffer (100µL/retina) containing 0.1% nonionic detergent Igepal CA630, 50 mmol/L HEPES, pH 7.3, 5 mmol/L EDTA, 150 mmol/L NaCl, β-glycerolphosphate (4 mg/mL), 100 mmol/L NaF, 1 mmol/L PMSF and 5 µL of orthovanadate. After centrifugation at 14 000 rpm the supernatants were kept for protein determination. 50 µg of protein were loaded onto the 4-20% gradient SDS-polyacrylamide gels (SDS-PAGE) under reducing condition. Proteins were electroblotted onto PVDF membrane in the presence of 48 mmol/L Tris-HCl (pH 6.8), 39 mmol/L glycine, 0.0037% SDS, and 20% methanol using semidry condition (Transblotting apparatus, Biorad). Membranes were stained with 0.5% ponceau S to check for protein transfer. After brief wash, membranes were blocked with 5% nonfat milk in 0.5% Tween-20 in TBS buffer for 1 hour at 4°C. Membranes were incubated with following primary antibodies at 4°C overnight: mouse anti-CD74 (1:500, Santa Cruz, sc-70782), rabbit anti-sEH (1:1000, Santa Cruz, sc-22344), rabbit anti-Notch1 (1:1000, Cell Signaling Technology^®^, #4380), Notch3 (1:1000, abcam, ab23426), Hes1 (1:1000, Cell Signaling Technology^®^, #11988) and GAPDH-HRP (1:2000, abcam, ab9385). Peroxidase conjugated anti-mouse (1:2000, Dako, P0447) and anti-rabbit (1:3000, Dako, P0448) antibodies were incubated for 1 hour and the immunoreactivity was visualized by enhanced chemiluminescence according to the manufacturer's protocol (Western Lighting Plus ECL, PerkinElmer). Fusion SL system (Peqlab) was used for all Western images and the densitometry was measured using Image J software. GAPDH served as the house keeping gene.

### RT-qPCR

Total RNA was isolated from retinal tissues using TRIZOL reagent. First strand cDNA was synthesized according to the manufacturer's instructions with the QuantiTect Reverse Transcription Kit (Qiagen, Hilden, Germany). A 20 µL real time PCR reaction volume was set up by mixing 10 µL universal PCR master mix, 6 µL AB primer (1:6 diluted), and 4 µL of diluted cDNA (1:10). Reactions were performed in a 96-well Micro Amp plate with the following steps: 50°C for 2 minutes, 95°C for 10 minutes, then 40 cycles of 95°C for 15 sec and 60°C for 1 minute per cycle. ABI 7100 PRISM sequence detector was used to measure fluorescent signals. Relative gene expression was calculated using the 2 (-∆∆Ct) method. Reference numbers of all primers used in this study were listed in Table 1.

*Ephx*1-4, gene expression levels were detected using SYBR Green (Absolute QPCR SYBR Green Mix; Thermo Fisher Scientific). The relative expression levels of *Ephx* genes were calculated using the 2-(-∆∆Ct) method with 18S RNA as a reference. The primer sequences used were listed in Table 2.

### Microglial culture and velocity *in vitro*

Mouse microglial cells (BV-2 cells) were cultured in DMEM medium containing 10% FCS (Gibco, Invitrogen), 100 U/mL penicillin, and 100 µg/mL streptomycin. Cells were seeded on DLL4 coated plates (500 ng/mL, R&D systems) to activate Notch signaling and BSA coated plates (0.1% in PBS) served as controls. After cells were treated with 3 μmol/L 19,20-EDP and 3 μmol/L 19,20-DHDP or 10 μmol/L DAPT in the present of sEH-I (10 μmol/L) for 24 h, the levels of Notch target genes Hey1 and Hes1 were determined by quantitative PCR. To determine microglia motility, cells were labeled with cell tracker green (Invitrogen) and seed on μ-slides (ibidi, Martinsried, Germany) coated with BSA or Dll4 and treated as above. Cells were incubated in an IncuCyte imaging system (Essen Bioscience) that took photographs automatically every 15 minutes. Cell movement was tracked manually and analyzed with Image J software (NIH, Bethesda, MD, USA). 200 cells were randomly selected and analyzed for each condition. The average cell mobility was expressed mean distance (µm) in each frame interval (15 minutes).

### Flow cytometry

Surface expression of CD74 on BV-2 cells was assessed by flow cytometry. Briefly, after treatment cells were suspended in 150 µL culture medium and blocked with Fc block (BD Pharmingen) for 20 minutes on ice. Then cells were incubated with FITC-CD74 (1:100, BD Pharmingen) in the dark at 4°C for 30 minutes. After washed three times with ice-cold PBS, cells were dissolved in 500 µL FACS buffer (BD Pharmingen) and analyzed on a BD FACSVerse flow cytometer (BD, Heidelberg, Germany).

### *Ex vivo* retinal explant culture

Eyes from one month old PKD rats were enucleated and immersed in ice-cold HBSS solution with 100U/mL penicillin and 100µg/mL streptomycin. Retinae were dissected under a steromicroscope and divided into four quadrants with four radial incisions. The explants were transferred onto tissue culture inserts (0.4 µm pore, Millipore) with ganglion cell side up and photoreceptor side down. The inserts were placed into the wells of a 6 well plate. A serum-free retinal explant medium (Neurobasal A, Invitrogen) supplemented with 2% B-27 (Invitrogen), 1% N-2 (Invitrogen), l-glutamine (0.8 mM), penicillin (100 U/mL), and streptomycin (100 μg/mL) was added to the bottom of the wells and 3 μL of medium was dropped on top of the retina to keep it moist. Retinal explant cultures were maintained in humidified incubators (37 °C, 5% CO_2_) and treated with solvent (0.03% DMSO), 3 μmol/L 19,20-EDP, 3 μmol/L 19,20-DHDP, or 10 µmol/L γ-secretase inhibitor DAPT in the presence of 10 µmol/L sEH-I. After 24 hours, retinae were fixed and stained using immunofluorescence. For quantification of 19,20-DHDP-induced microglial activation, retina explants were co-stained with antibodies of mouse anti-rat CD11b^+^ (1:100 dilution, MCA275G, AbD SeroTec) and goat anti-rat CD74^+^ (1:100 dilution, SC-5438, Sant Cruz). The secondary antibodies goat anti-mouse Alexa Fluor^®^ 488 (Invitrogen, A21131, 1:200 dilution) and donkey anti-goat Alexa Fluor 555^®^ (Invitrogen, A21432, 1:200 dilution) were incubated at room temperature for one hour. Images were taken using a Leica TCS SP2 confocal microscope. Ten randomly selected fields were quantified for activated CD11b^+^/CD74^+^ microglial cells.

### Statistics

Data are expressed as mean ± SEM. Statistical evaluation (GraphPad Prism 6 software) was performed using Student's t test for unpaired data, one-way ANOVA or two-way ANOVA where appropriate. Values of P<0.05 were considered statistically significant.

## Results

### sEH expression and activity are increased in PKD rat retinae

**Figure 1:**
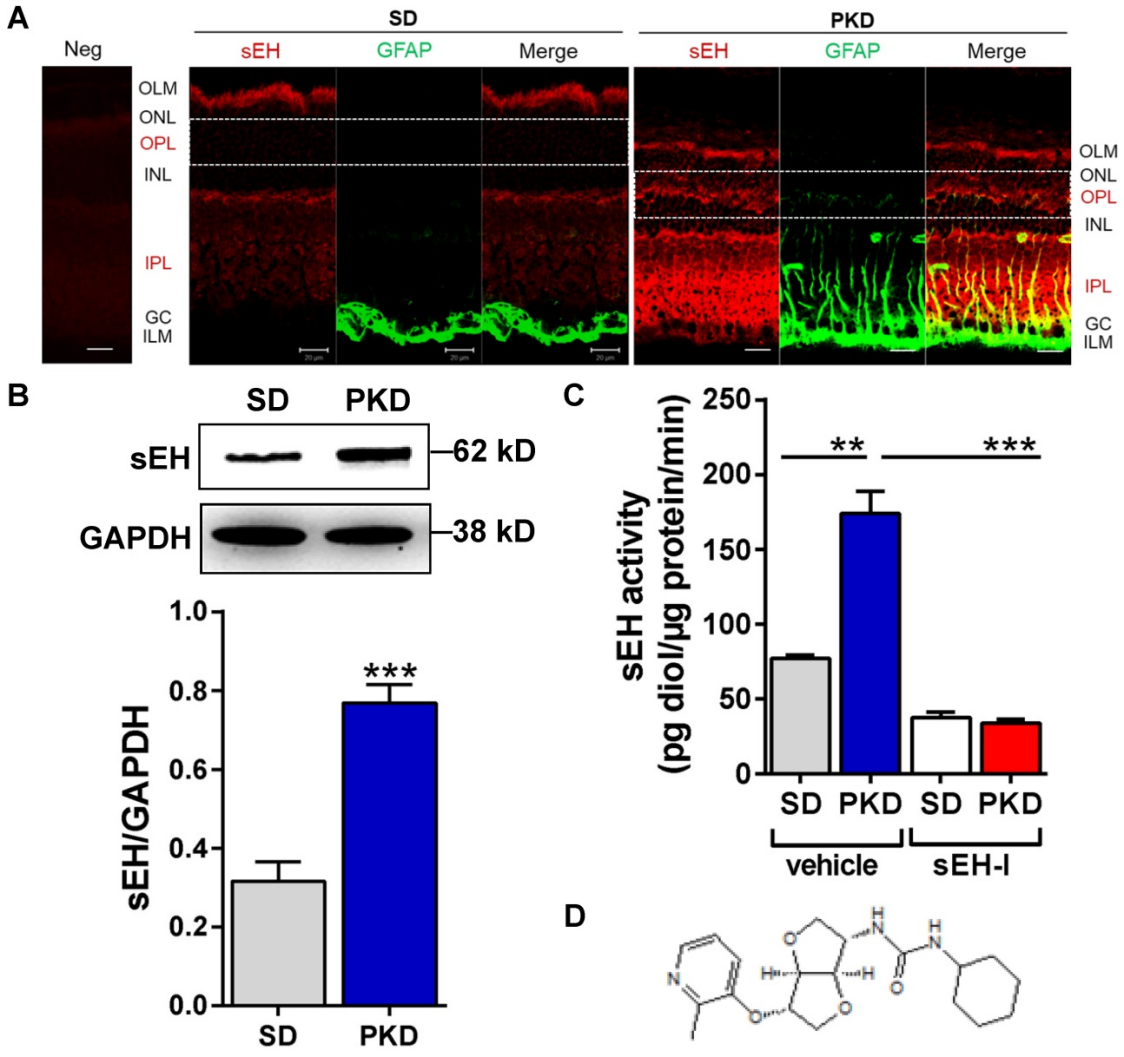
** Soluble epoxide hydrolase (sEH) expression and activity are increased and Sar5399 effectively restrains its activity in retinae from PKD rats. (A)** sEH (red) and glial fibrillary acidic protein (GFAP, green) expression was assessed by confocal microscopy of retinal cryo-sections (6 µm) from 3 month old Sprague Dawley (SD) and polycystic kidney disease (PKD) rats; bar = 20 µm. On the far left a negative control (Neg) using rabbit IgG was included. ILM: inner limiting membrane. GC, ganglion cell layer; IPL, inner plexiform layer; INL, inner nuclear layer; OPL, outer plexiform layer; ONL, outer nuclear layer; OLM, outer limiting membrane. sEH expression was labeled in red color and particularly observed in IPL and OPL of PKD rats. Comparable results were obtained in retinae from three additional animals.** (B)** Representative blot and quantification of sEH protein expression in retinae from SD and PKD rats; n = 5; ***P < 0.001 (unpaired t-test, two tailed). **(C)** Retinal sEH activity (pg diol/µg protein/minute) in retinae from SD and PKD rats treated with vehicle or Sar5399 (sEH-I; 30 mg/kg, 4 weeks); n = 5; **P < 0.01, ***P < 0.001 (two-way ANOVA with Tukey's multiple comparisons test). **(D)** Chemical structure of Sar5399.

The localization and expression of sEH in control SD and PKD rats was determined by immunofluorescence microscopy. Similar to previous reports in mice and humans 26, 28, the sEH was detected in SD rat retina in the inner and outer plexiform layers (IPL and OPL) and in the outer limiting membrane (OLM) where Müller cells are located (**Figure 1A**). Glial fibrillary acidic protein (GFAP) expression was confined to astrocytes and the end feet of Müller cells in the ganglion cell layer (GC) in SD rat retinae. However, the GFAP signal was markedly increased in Müller glial cells and inner plexiform layer (IPL) of PKD retinae, indicating their activation (**Figure 1A**). sEH expression was also markedly greater in activated Müller glia in PKD rats and the immunofluorescence data were confirmed by Western blotting (**Figure 1B**), and a significant increase in sEH enzymatic activity (**Figure 1C**). The sEH inhibitor (sEH-I) Sar5399 (**Figure 1D**) effectively decreased sEH activity in the SD and PKD rat retinae.

### sEH inhibition reduces generation of diol 19,20-DHDP and prevents retinal vasoregression

**Figure 2:**
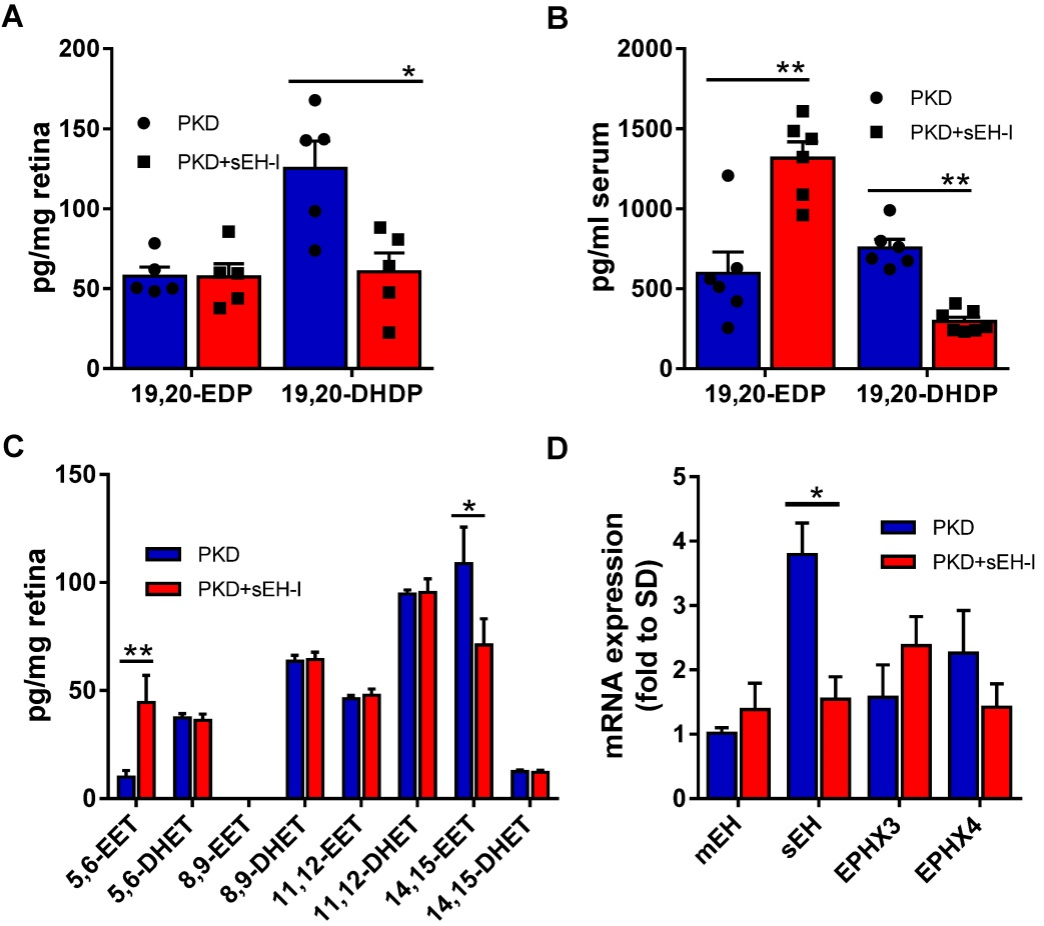
** sEH inhibition alters epoxide, diol and EPHX levels. (A)** Levels of 19,20-EDP and 19,20-DHDP in retinae from PKD rats treated with vehicle or sEH-I Sar5399 (30 mg/kg) for four weeks; n = 5; *P < 0.05 (two-way ANOVA with Sidak's multiple comparisons test). **(B)** Levels of 19,20-EDP and 19,20-DHDP in serum from vehicle- and sEH-I-treated PKD rats; n = 6; **P < 0.01 (two-way ANOVA with Sidak's multiple comparisons test). **(C)** Effect of sEH inhibition on levels of arachidonic acid-derived EET and DHET levels in retinae from PKD rats; n = 5; **P < 0.01 (two-way ANOVA with Sidak's multiple comparisons test). **(D)** Relative mRNA expression of epoxide hydrolase isozymes in SD rats, as well as vehicle- and sEH-I treated PKD. mEH = microsomal epoxide hydrolase (or *EPHX1*); sEH, (*EPHX2*); *EPHX3* and *EPHX4*; n = 3; *P < 0.05, ***P < 0.001 (two-way ANOVA with Tukey's multiple comparisons test).

To determine whether the increase in sEH expression and activity could be linked to vasoregression, PKD rats were treated with a sEH inhibitor. Four weeks treatment with the sEH inhibitor significantly decreased retinal (**Figure 2A**) and serum (**Figure 2B**) levels of the sEH product 19,20-DHDP. Consistent with the fact that DHA levels are higher than those of arachidonic acid in the retina 35, higher levels of the DHA-derived lipids, 19,20-EDP and 19,20-DHDP, were detected in the retina than the epoxides (epoxyeicosatrienoic acids or EETs) and diols (dihydroxyeicosatrienoic acid or DHETs) derived from arachidonic acid (**Figure 2A,C**). While the accumulation of 19,20-EDP was not changed in retinal samples, levels did increase significantly in serum (**Figure 2A,B**). Of the other CYP- and sEH-derived PUFA metabolites assessed, sEH inhibition resulted in an increase in the amount of 5,6-EET and but a decrease in 14,15-EET (**Figure 2C**). The effects on 5,6-EET were unexpected as this epoxide is a poor substrate for the sEH and is more rapidly metabolized by cyclooxygenase 36. The sEH is not the only epoxide hydrolase isoform. The expression of the sEH (gene = *Ephx2*) was increased in retinae from PKD rats and sEH-inhibitor reduced mRNA expression of *Ephx2*, while the expressions of the microsomal epoxide hydrolase (mEH, *Ephx1*), *Ephx3* and *Ephx4* were not significantly affected (**Figure 2D**).

**Figure 3:**
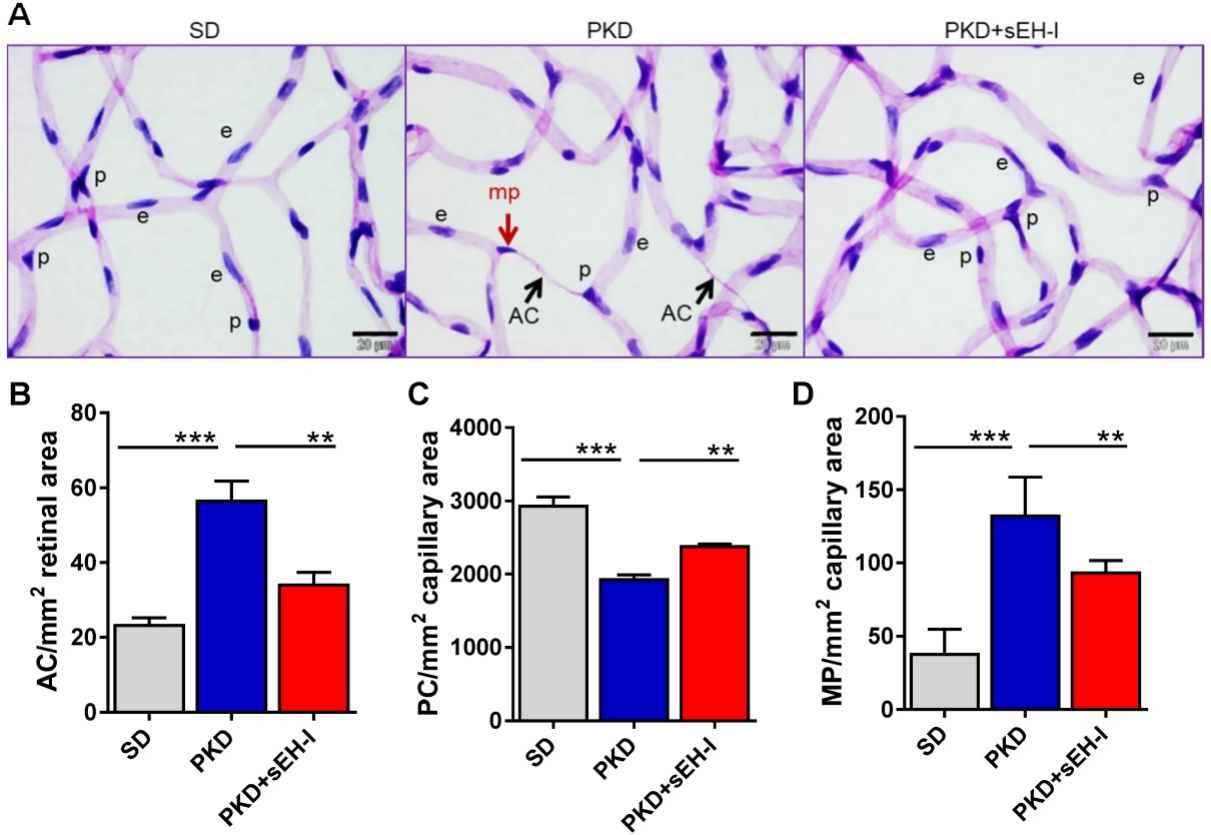
** sEH inhibition reduces pericyte loss and ameliorates vasoregression.** Retinal morphometry was measured in SD and PKD rats with and without sEH inhibition. Four weeks old SD and PKD rats were treated with vehicle or sEH-I (30 mg/kg) for four weeks. **(A)** PAS and haematoxylin stained retinal digest preparations. Black arrows indicate acellular capillaries (AC) and the red arrow indicates a migrating pericyte (mp); p = pericyte, e = endothelial cell, bar = 20 µm. Comparable results were obtained in retinae from five additional animals. **(B)** Quantification of acellular capillaries (AC/mm^2^ retinal area). **(C)** Quantification of pericytes (PC/mm^2^ capillary area). **(D)** Quantification of migrating pericytes (MP/mm^2^ capillary area). **(B-D)** n=5, **P < 0.01, ***P < 0.001 (one-way ANOVA with Tukey's multiple comparisons test).

Retinal digest preparations were used to compare the retinal vasculature from SD and PKD rats (**Figure 3A**). Acellular capillaries were detected in retinae from PKD rats but absent in samples from SD rats. The administration of the sEH inhibitor to the PKD rats largely rescued the phenomena and significantly attenuated the occurrence of acellular capillaries (**Figure 3B**). Pericyte numbers were also significantly lower in retinae from PKD versus SD rats and sEH inhibition increased pericyte numbers in the PKD rats (**Figure 3C**). Pericyte migration, characterized by altered nuclear shape, capillary contact interface and orientation towards the pericapillary interstitium 33, 34, was also substantially increased in PKD rats, and markedly reduced by sEH treatment (**Figure 3D**).

### sEH inhibition restrains microglia activation

CD74 is a receptor for macrophage migration factor 37 and the invariant chain of class II major histocompatibility complex 38. It is also a microglia activation marker in the retina and CD74 was expressed on microglia in PKD retinae in close vicinity to “degenerated” capillaries suggesting a link between microglial activation and vasoregression. Therefore, the pan-microglia marker, CD11b, was used together with CD74 15 to assess the pericapillary inflammatory potential in the retinal vasculature.

**Figure 4:**
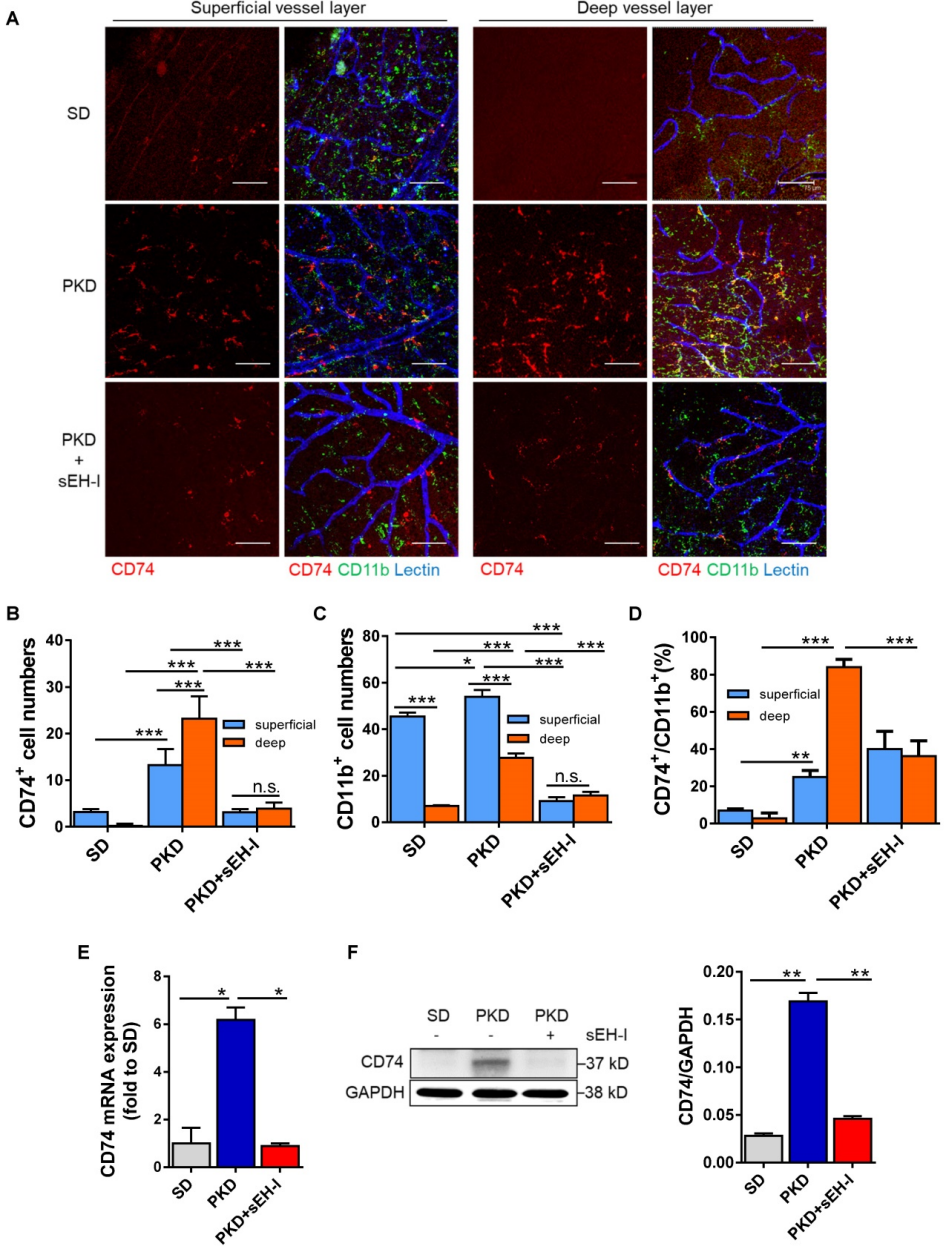
** sEH inhibition reduces microglial recruitment and CD74 expression. (A-D) Visualization and quantification of microglia. (A)** Whole mount retinal preparations (400x magnification) showing microglia (CD11b^+^/CD74^+^) in the superficial and deep layer of retinae from SD rats, and vehicle or sEH-I-treated PKD rats. Scale bar = 75 µm, CD11b = green, CD74 = red, lectin = blue. **(B)** Quantification of CD74^+^ cells in the superficial and deep layers of retinae from SD rats, as well as vehicle- and sEH-I-treated PKD rats. **(C)** Quantification of CD11b^+^ cells in in the superficial and deep layers of retinae from SD, vehicle or sEH-I-treated PKD rats. **(D)** Percentage of CD74^+^ microglia in the CD11b^+^ cells in the superficial and deep layers of retinae. **(B-D)** n = 5; *P < 0.05, ** *P* < 0.01, ***P < 0.001, (two-way ANOVA with Sidak's multiple comparisons test) ns= not significant. **(E)** Relative CD74 mRNA expression in retinae; n = 3; *P < 0.05 (one-way ANOVA with Tukey's multiple comparisons test). **(F)** Retinal CD74 protein levels; n = 5; **P < 0.01 (one-way ANOVA with Tukey's multiple comparisons test).

In retinae from SD rats, there were scattered CD74^+^ microglia in the superficial capillary layer but CD74^+^ cells were almost undetectable in the deep capillary layer (**Figure 4A,B**). In the same samples, CD11b^+^ cells were predominantly located in the superficial capillary layer of the retinal vasculature (**Figure 4C**). Only approximately 7% of the cells were double positive for CD11b and CD74 (**Figure 4D**), and again only a few microglial cells were detected in the deep capillary layers. In PKD retinae, the numbers of CD74^+^ and CD11b^+^ microglia cells were increased in both layers but the most pronounced change was detected in the deep capillary layer where 84% were CD11b^+^ and CD74^+^, indicating increased microglial activation. In the PKD rats treated with the sEH inhibitor, microglial abundance and microglial activation were significantly attenuated compared with the vehicle-treated PKD rats. This implied that sEH inhibition not only reduced microglia activation but also attenuated microglia recruitment and migration. The histological changes in microglial activation could be confirmed at the level of CD74 mRNA (**Figure 4E**) and protein expression in retinal lysates (**Figure 4F**).

### 19,20-DHDP suppresses Notch signaling and promotes microglia activation and migration

**Figure 5:**
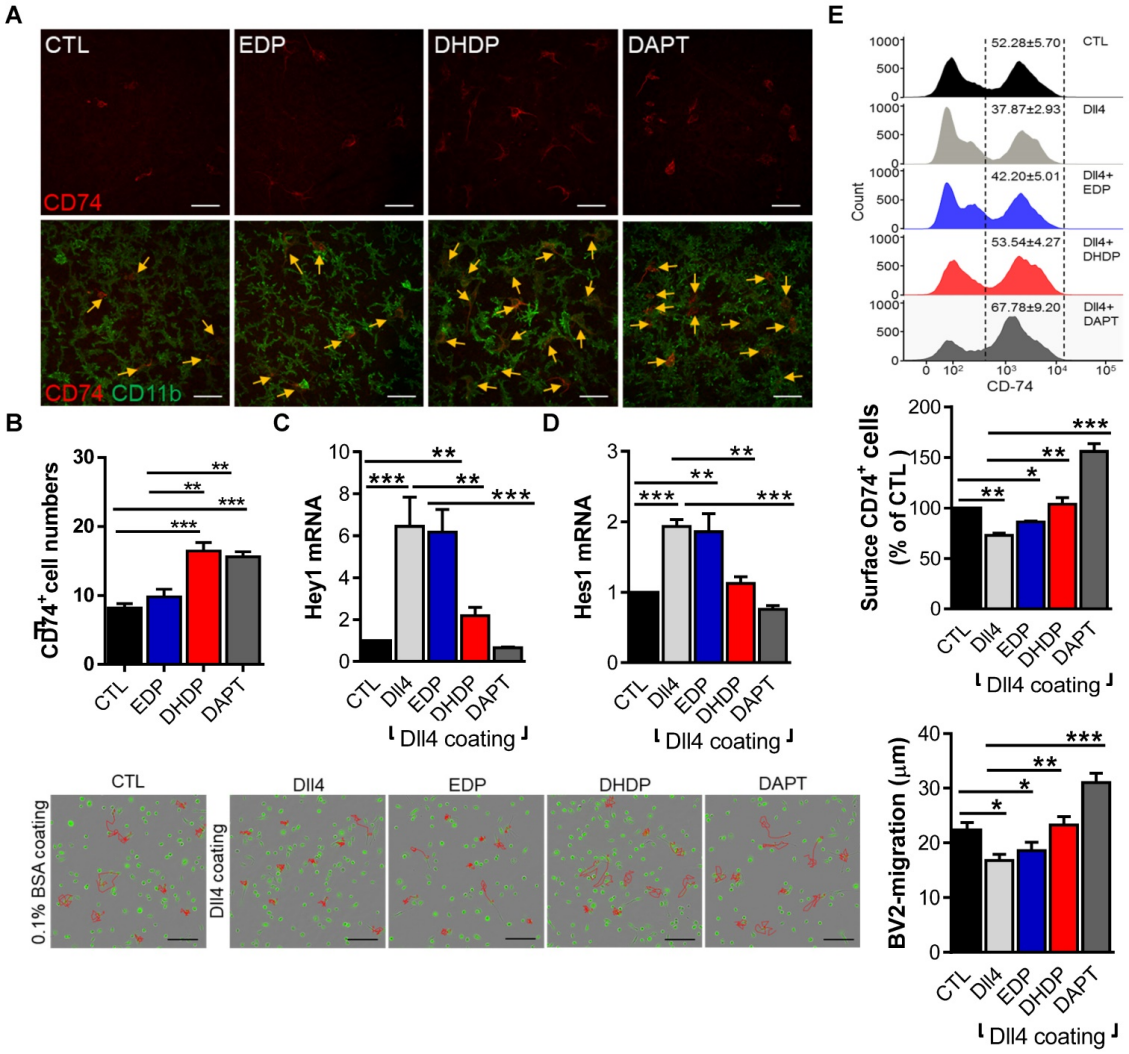
** 19,20-EDP and 19,20-DHDP impact Notch signaling and microglial activation. (A-B)** For *ex vivo* retinal explants cultures, retinae from one-month old PKD rats were incubated for 24 hours in 10 µmol/L sEH-I with 0.03% DMSO (CTL), 3 µmol/L EDP, 3 µmol/L DHDP or 10 µmol/L DAPT. **(A)** CD74+ (red) and CD11b+ (green) retinal microglia, the yellow arrows indicate CD74^+^ microglia; bar = 50 µm. **(B)** Quantification of CD74^+^ microglial cells; n = 4, **P < 0.01, ***P < 0.001 (one-way ANOVA with Turkey's multiple comparison test). **(C-F)** BV-2 cells were seeded on plates coated with 0.1% BSA (CTL) or Dll4 (500 ng/mL) in the continued presence of sEH-I (10 µmol/L) and treated for 24 hours with 19,20-EDP (3 μmol/L), 19,20-DHDP (3 μmol/L) or γ-secretase inhibitor (DAPT,10 μmol/L). **(C-D)** RT-qPCR analysis of Hey1 (C) and Hes1 (D) mRNA in retinal explants. **(E)** Surface expression of CD74 on BV-2 microglial cells. **(F)** Mobility tracing of microglia (red lines) over a period of 4 hours. The average mobility was calculated as μm/frame (15 minutes intervals); bar = 200 µm. **(C-F)** n = 4, *P < 0.05, **P < 0.01, ***P < 0.001 (one-way ANOVA with Dunnett's multiple comparison test).

Given the important role of Notch signaling in microglia activation 27, and the fact that 19,20-DHDP inhibits Notch signaling 26, we hypothesized that the metabolite most affected by sEH inhibition i.e. 19,20-DHDP, could actively induce microglia activation. To address this hypothesis, *ex vivo* retinal explants were maintained in culture in the continual presence of an sEH inhibitor and absence and presence of 19,20-DHDP and the γ-secretase inhibitor DAPT. Both 19,20-DHDP and DAPT induced microglial activation and promoted CD74^+^ microglial cell recruitment, while the sEH substrate 19,20-EDP did not (**Figure 5A,B**). Furthermore, in cultured murine microglial (BV-2) cells that expressed CD74, the Notch ligand; delta-like ligand 4 (Dll4) elicited Notch activation and an increase in Hey1 and Hes1 expression (**Figure 5C,D**). The sEH substrate 19,20-EDP failed to affect this response, while 19,20-DHDP prevented Notch activation. The extent of the inhibition by 19,20-DHDP was similar to that of the γ-secretase inhibitor DAPT. In parallel, Dll4-induced Notch activation significantly decreased CD74 expression (**Figure 5E**), and this effect was reversed by 19,20-DHDP. Given that sEH inhibition reduced microglial activation and recruitment in PKD rats the effects of Notch activation on microglial migration were assessed. While Notch activation decreased microglia migration, the effect was reversed in the presence of 19,20-DHDP but not 19,20-EDP (**Figure 5F**). These findings indicate that lipid signaling through modulation of Notch activation largely controls CD74 surface expression and microglia activation/migration in the retina.

### sEH inhibition increases Notch signaling and reduces immune cytokines expression

**Figure 6:**
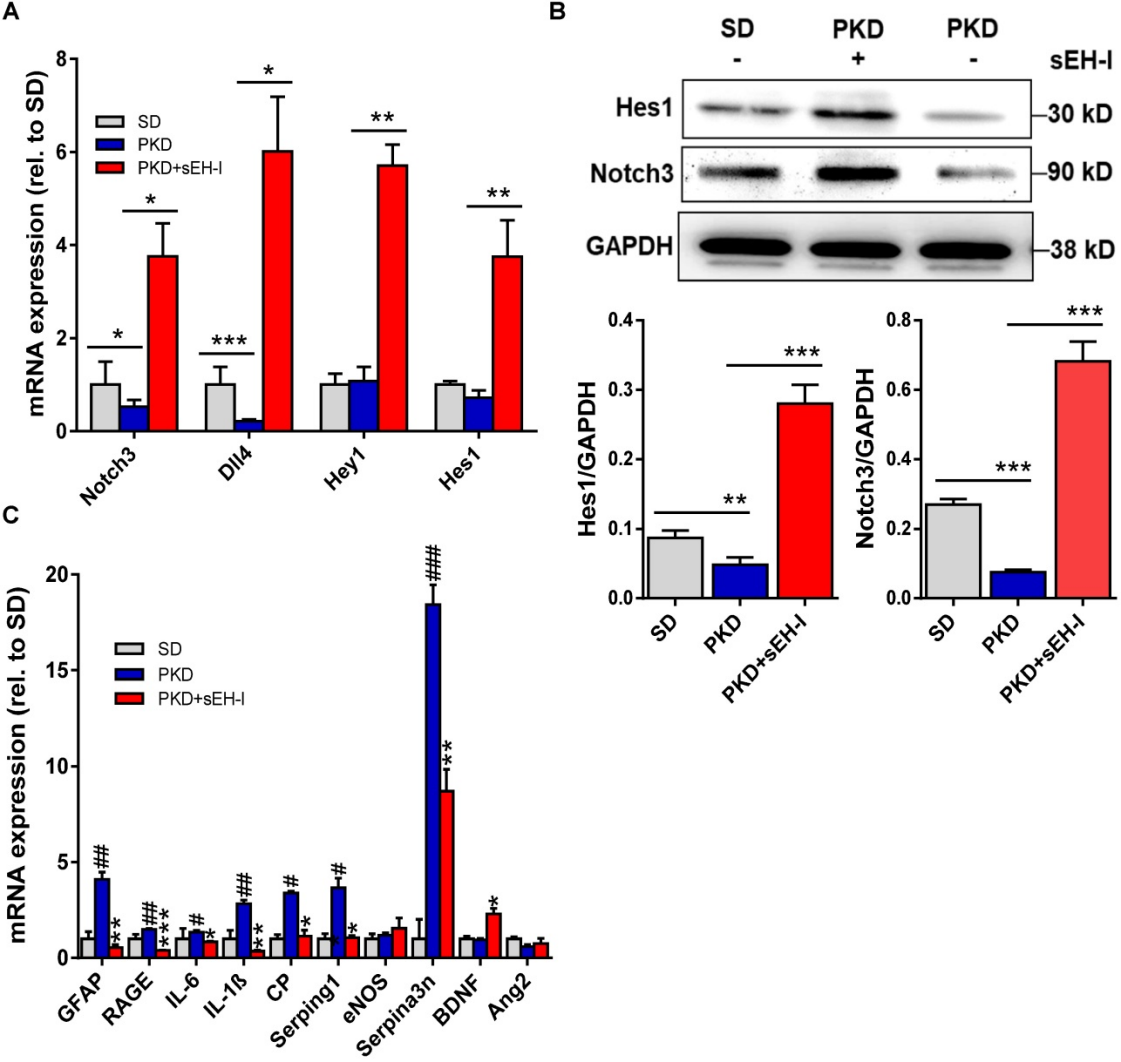
** sEH inhibition modulates Notch and affects retinal gene expression. (A)** mRNA expression of Notch signaling genes in SD rats, and vehicle treated or sEH-I-treated PKD rats; n = 4; *P < 0.05, **P < 0.01, ***P < 0.001 (one-way ANOVA with Dunnett's multiple comparisons test). **(B)** Immunoblot and quantification of Hes1 and Notch3 expression in retinae from SD and PKD rats with (+) and without (-) sEH-I treatment; n = 5; **P <0.01 and ***P < 0.001 (one-way ANOVA with Tukey's multiple comparisons test). **(C)** Genes potentially associated with retinopathy and vasoregression; n = 4; *P < 0.05, **P < 0.01, ***P < 0.001 versus vehicle treated PKD; ^#^*P* < 0.05, ^##^*P* < 0.01, ^###^*P* < 0.001 versus SD rats (one-way ANOVA with Dunnett's multiple comparisons test).

To determine the impact of sEH inhibition on glial activation and the immune responses, the retinal expression of relevant genes was determined. Compared to retinae from SD rats, retinae from PKD rats demonstrated decreased expression of Notch 3 and Dll4 (**Figure 6A,B**). sEH inhibition resulted in a marked activation of the Notch signaling pathway and increased the expression of the receptor Notch 3 and the agonist Dll4, as well as the downstream Notch-regulated genes; Hey1 and Hes1. In the same samples significant differences in the expression of GFAP, the receptor for advanced glycation end-product (RAGE), interleukin (IL)-6, IL-1β, ceruloplasmin (CP), serpin g1 and serpin a3n were detected between retinae from SD and PKD rats (**Figure 6C**). sEH inhibition prevented the increase in all of these activation markers in PKD retinae at the same time as increasing the expression of the Müller cell derived neuroprotective protein brain-derived neurotrophic factor 39-41. Importantly, the expression of angiopoietin 2 (Ang-2), which has frequently been linked with vasoregression in other models 33, 42, was not increased in PKD retinae (versus SD) and was unaffected by sEH inhibition. These observations support a strong anti-inflammatory effect of sEH inhibitor treatment in PKD rats and makes a strong link to neurodegeneration-induced vasoregression.

## Discussion

**Figure 7:**
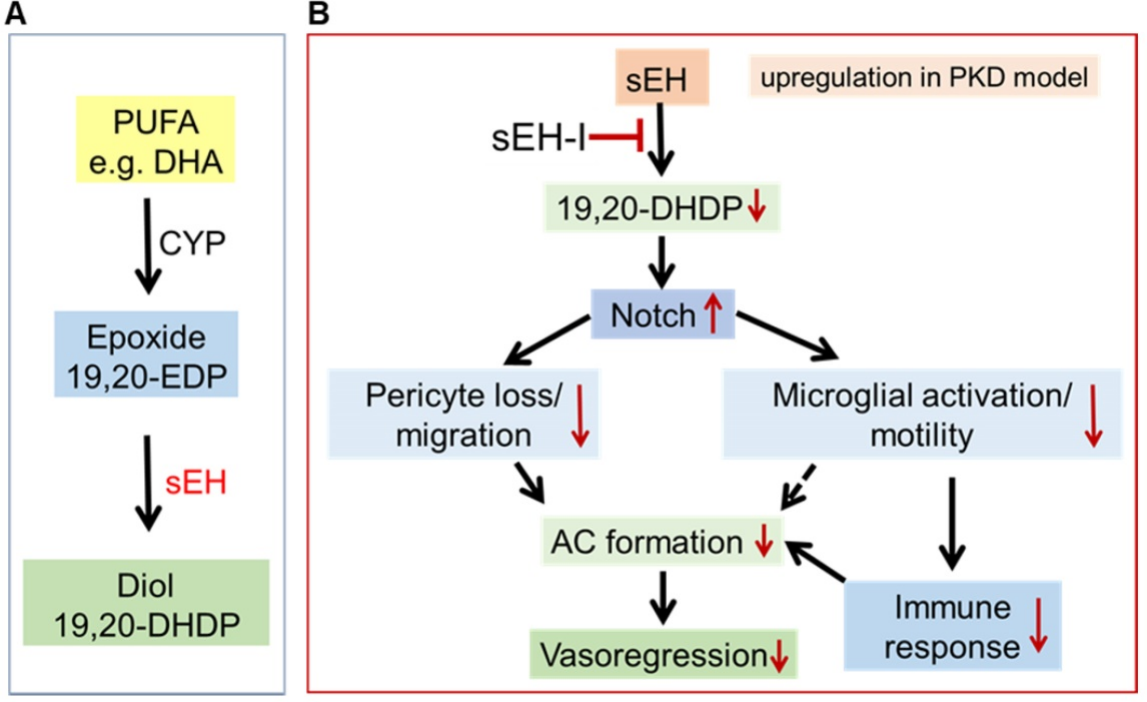
** Proposed mechanism by which sEH inhibition affects the neurovascular unit in the degenerating retina. (A)** Scheme showing the metabolism of the polyunsaturated fatty acid (PUFA), docosahexaenoic acid (DHA), by CYP enzymes to generate the epoxide 19,20-EDP, which is in turn metabolized by the sEH to generate the diol 19,20-DHDP. **(B)** In the PKD retina, increased activity of the sEH (in Müller glia and astrocytes) results in the generation of 19,20-DHDP which inhibits Notch signaling and activates microglia to induce vasoregression. Treatment with an sEH-I reduces formation of 19,20-DHDP and upregulates Notch signaling, which subsequently reduces both pericyte loss/migration and microglial activation/motility. This ameliorates vasoregression and the formation of acellular capillaries (AC) through prevented pericyte loss and reduced activity of inflammatory microglia.

The results of the present investigation revealed that a significant increase in the expression of sEH in PKD rat retinae leads to an increase in the production of 19,20-DHDP. The increased levels of 19,20-DHDP triggered pericyte migration away from the vasculature and significantly affected the Notch signaling pathway to increase microglial activation, which has also been linked with destabilized retinal capillaries 43-46. Importantly, sEH inhibition prevented the vasoregression detected in PKD rats, most likely by attenuating the activation of microglia and their subsequent immune response via the upregulation of the Notch signaling pathway (see **Figure 7**).

Pericyte migration and loss are primarily considered as pathogenic in diabetic retinopathy 3, 47. The PKD model also displays pericyte loss to a level that is comparable to that observed in the diabetic rat retina 5. Several mechanisms have been proposed to account for pericyte recruitment/loss and migration, with Ang-2 being the factor that has received the most attention 33, 42, 48-50. However, Ang-2 levels were comparable in the SD and PKD retinae and were unaffected by sEH inhibition. Although not studied in detail here, additional hypoxia-stimulated growth factors have been implicated in retinal vessel survival and maturation, including vascular endothelial growth factor and platelet-derived growth factor 51. However, neither of these proteins were affected in a mouse model of non-proliferative diabetic retinopathy or by sEH inhibition 5, 28.

The lipid mediator in focus of the present investigation was 19,20-DHDP. This PUFA diol is synthesized by the sEH from 19,20-EDP which is generated from DHA by the action of CYP enzymes. Several CYP enzymes are expressed in the retina, and the murine Cyp2c44, which is closely related to the human CYP2C8 and CYP2C9 isoforms 52, is expressed in Müller glia and its deletion has been linked to retinal angiogenesis via a mechanism linked to attenuated Notch signaling 53. Thus, the loss of Cyp2c44 elicited exactly the opposite phenotype to the loss of the sEH where the defect in vasculogenesis was attributed to activation of the Notch signaling cascade 26. Interestingly, Cyp2c protein expression in retinal monocytes/macrophages has also been linked to inflammation and pathologic neovascularization 54. CYP enzyme expression was however not the focus of the current investigation as PUFA epoxide levels did not differ markedly in the SD and PKD retinae, with one exception i.e. 5,6-EET. Rather, we focused on the sEH as its expression was statistically increased in the PKD retinae and the 19,20-diol of DHA was present in much higher concentrations than all other lipid mediators. One additional epoxide hydrolase was slightly (non-significantly) increased in the PKD retinae i.e. Ephx4, however almost nothing is known about the actions of this enzyme or its catalytic profile. Moreover, the clear positive effects of sEH inhibition on vascular regression and microglia activation were paralleled by a robust decrease in 19,20-DHDP levels. Importantly, high concentrations of 19,20-DHDP have previously been linked with vasoregression and diabetic retinopathy 28, and reduced levels of 19,20-DHDP have been attributed vasoprotective and anti-inflammatory properties 55. Ideally, a direct link between these events could be demonstrated by the injection of 19,20-DHDP into the retina. However, the repeated injections required for the phenotype to develop over several months is not technically feasible. The over expression of the sEH in Müller glia cells in mice, however, has been shown to rapidly elicit pericyte loss and vasoregression 28. We have previously used cell-specific sEH knockout models to show the expression of the sEH in the murine retina is highest in Müller cells but that astrocytes also express the enzyme. While the Müller cell sEH had the most impact on physiological angiogenesis and diabetic retinopathy astrocyte-derived products of the sEH can affect the retinal vascular phenotype in a model of retinopathy of prematurity 56. In the PKD rat the most marked changes in sEH expression were in cell that spanned the entire retina and thus more closely correspond to Müller cells, however we cannot exclude that the expression of the sEH in the PKD rat was also increased in astrocytes.

Müller cell-derived 19,20-DHDP is an effective inhibitor of the γ-secretase and thus of Notch signaling 26. The fact that both 19,20-DHDP and the γ-secretase inhibitor, DAPT, elicited comparable effects on CD74 as well as Hes1 and Hey1 expression hints that altered activation of the Notch signaling pathway could account for the observed effects on vasoregression and microglia activation. This is of relevance as Notch signaling is important to preserve vessel maturity and integrity as well as to regulate pericyte behavior, including the recruitment of mural cells in the retina 57-59. Notch3 seems to be particularly important for pericyte coverage in retinal vessels 59, 60, and increased activation of Notch signaling in a disease state, i.e. when pericyte loss has already occurred, improves pericyte coverage 61. Thus, as a consequence of the decrease in the levels of an endogenous Notch inhibitor, i.e., 19,20-DHDP, the de-repression of Notch signaling may prevent vasoregression. Of note, our data indicated a clear upregulation of Notch and its downstream transcription factors in PKD rats treated with the sEH inhibitor - that were even higher than those detected in retinae from SD rats. It is tempting to speculate that an excess activation of Notch activity could lead to an overgrowth of retinal capillaries with pericytes. However, there was no evidence of retinal capillary pericyte overgrowth under physiological conditions. Rather, by comparing the consequences of sEH expression and 19,20-DHDP on physiological angiogenesis and in diabetic retinopathy it seems that low concentrations of the DHA-derived diol promotes angiogenesis and vascular development, while higher concentrations do the opposite and interfere with cholesterol-binding proteins in membranes to promote pericyte loss and vasoregression 62. Altered Notch signaling results in microglial activation 63, implying that the same signaling pathway can concomitantly regulate microglia activity and vasoregression.

The role of inflammation has been widely acknowledged in the pathogenesis of vasoregression, spanning from retinal aging to diabetic retinopathy and neurodegenerative diseases 64-66. The common denominator is the microglial overproduction and secretion of inflammatory cytokines which activate and damage endothelial cells and pericytes. This is important as sEH inhibitors have been attributed anti-inflammatory effects in numerous animal models, including the retina 29. Reducing the numbers of retinal CD74^+^ microglial cells by sEH inhibition lowered inflammatory and immune response cytokines including IL-1β, CP, Serping1 and Serpinga3n, which are important immune components 67, 68. Under physiological conditions, microglial cells confer immuno-protection against transient deleterious stimuli 66. Under disease conditions, such as in the PKD model, photoreceptor-degeneration induced chronic activation of microglia involves degenerative and immune responses as key pathologies 69, 70. Alleviation of both microglial pro-migratory and pro-inflammatory properties by sEH inhibition is likely to affect vascular damage simultaneously, because of a parallel effect on Notch mediated pericyte stabilization and a Notch mediated inhibition of microglial activation and cytokine-induced endothelial damage. Therefore, microglial activation was also closely associated with pericyte loss and vasoregression in the PKD model.

Taken together, data from this study highlight the importance of sEH expression by Müller glia cells to the homeostasis of the retina and in particular the importance of lipid mediators in communication within the neurovascular unit to affect both inflammation and vasoregression. Given that sEH inhibition results in anti-inflammatory effects and protects against vasoregression, it will be interesting to translate these findings into other preclinical models of retinal degeneration and into defined clinical disease settings.

## Figures and Tables

**Table 1:** List of primers used in this study

Primers for rat	
Gene name	Reference number
*CD74*	Rn00565062_m1
*Notch3*	Rn00571731_m1
*Dll4*	Rn01512886_m1
*Hey1*	Rn00468865_m1
*Hes1*	Rn00577565_g1
*GFAP*	Rn00566603_m1
*RAGE/Ager*	Rn00584249_m1
*IL-6*	Rn00561420_m1
*IL-1β*	Rn00580432_m1
*CP*	Rn00561049_m1
*eNOS*	Rn02132634_s1
*Serpina3n*	Rn 00755832_mH
*BDNF*	Rn01484924_m1
*Ang2*	Rn01756774_m1
*GAPDH*	Rn99999916_s1
**Primers for mouse BV-2 cells**	
Gene name	Reference number
*Hey1*	Mm00468864_g1
*Hes1*	Mm01342805_m1
*GAPDH*	Mm99999915_g1

All primers were from Thermo Fisher Scientific. CD74, major histocompatibility complex, class II invariant chain; Notch3, notch receptor 3; Dll4, delta like ligand 4; Hey1, hairy and enhancer of split regulated with YRPW motif protein-1; Hes1, hairy and enhancer of split-1; GFAP, glial fibrillary acidic protein; RAGE, receptor for advanced glycation end products; IL-6, interleukin-6; IL-1β, interleukin-1 β; CP, ceruloplasmin; Serping1, serpin family G member 1; eNOS, endothelial nitric synthase; Serpina3n, serine protease inhibitor A3N; BDNF, brain-derived neurotrophic factor; Ang2, angiopoietin 2; GAPDH*,* Glyceraldehyde 3-phosphate dehydrogenase.

**Table 2:** Primer sequences for *Ephx* genes

genes	*forward*	*reverse*
*18S RNA*	5′-CTTTGGTCGCTCGCTCCTC-3′	5′-CTGACCGG-GTTGGTTTTGAT-3′
*Ephx1*	5′-GGAGAGTGGAGAAACTGCACA-3′	5′-TGAAGCCATAGTGGAAGCGG-3′
*Ephx2*	5′-GAGGGACCCACTGAGCAACTCATGA-3′	5′-ATGGGGGACACCTCAGGATTTGGTG-3′
*Ephx3*	5′-ATAGCCCCTCTGATGCTCCA-3′	5′-TGGCGGGTTGAGTATTCTTGG-3′
*Ephx4*	5′-ACGTGCGCATCAAGGACTC-3′	5′-GCATCGGACTCTCCGTAACC-3′

## Abbreviations

CD11b: cluster of differentiation 11b
CD74: cluster of differentiation 74
DHA: docosahexaenoic acid
DHDP: dihydroxydocosapentaenoic acid
DHET: dihydroxyeicosatrienoic acid
Dll4: delta-like ligand 4
EDP: epoxydocosapentaenoic acid
EET: epoxyeicosatrienoic acid
GC: ganglion cell
ILM: inner limiting membrane
INL: inner nuclear layer
IPL: inner plexiform layer
OLM: outer limiting membrane
ONL: outer nuclear layer
OPL: outer plexiform layer
PKD: polycystic kidney disease
SD: Sprague Dawley
sEH: soluble epoxide hydrolase
sEH-I: soluble epoxide hydrolase inhibitor
